# Assessing the Relationship between the Enhanced Gait Variability Index and Falls in Individuals with Parkinson's Disease

**DOI:** 10.1155/2020/5813049

**Published:** 2020-02-07

**Authors:** Abigail C. Schmitt, Sidney T. Baudendistel, Michaela S. Fallon, Jaimie A. Roper, Chris J. Hass

**Affiliations:** ^1^Applied Neuromechanics Laboratory, Department of Applied Physiology and Kinesiology, University of Florida, 1864 Stadium Road, Gainesville, FL 32611, USA; ^2^Locomotor and Movement Control Laboratory, School of Kinesiology, Auburn University, 301 Wire Road, Auburn, AL 36849, USA

## Abstract

Gait impairment and increased gait variability are common among individuals with Parkinson's disease (PD) and have been associated with increased risk for falls. The development of composite scores has gained interest to aggregate multiple aspects of gait into a single metric. The Enhanced Gait Variability Index (EGVI) was developed to compare an individual's gait variability to the amount of variability in a healthy population, yet the EGVI's individual parts may also provide important information that may be lost in this conversion. We sought to contrast individual gait measures as predictors of fall frequency and the EGVI as a single predictor of fall frequency in individuals with PD. 273 patients (189M, 84F; 68 ± 10 yrs) with idiopathic PD walked over an instrumented walkway and reported fall frequency over three months (never, rarely, monthly, weekly, or daily). The predictive ability of gait velocity, step length, step time, stance time, and single support time and the EGVI was assessed using regression techniques to predict fall frequency. The EGVI explained 15.1% of the variance in fall frequency (*p* < 0.001, *r* = 0.389). Although the regression using the combined spatiotemporal measures to predict fall frequency was significant (*p*=0.002, *r* = 0.264), none of the components reached significance (gait velocity: *p*=0.640, step length: *p*=0.900, step time: *p*=0.525, stance time: *p*=0.532, single support time: *p*=0.480). The EGVI is a better predictor of fall frequency in persons with PD than its individual spatiotemporal components. Patients who fall more frequently have more variable gait, based on the interpretation of the EGVI. While the EGVI provides an objective measure of gait variability with some ability to predict fall frequency, full clinical interpretations and applications are currently unknown.

## 1. Introduction

Impairment of walking abilities is among the most disabling symptoms associated with Parkinson's disease [1]. Both pace and rhythm are affected leading to the hallmark features of Parkinsonian gait: slow, shuffling steps. Further, individuals with PD spend more time in the double support phase of gait and adopt a wider base of support, perhaps in an effort to mitigate balance deficits [2, 3]. In addition to bradykinesia and balance impairments, a decreased ability to maintain a steady gait pattern (i.e., increased step-to-step variability), is commonly observed [4, 5]. These deficits in spatiotemporal parameters and increased gait variability become more pronounced with disease progression, leading to pronounced disability [4, 6]. Increased gait variability in individuals with PD is likely an indicator of deterioration within the motor control system [3, 4, 7]. Indeed, a variability of spatiotemporal parameters, such as stride time, has been related to increased incidence of falls in individuals with PD [7]. Falls are, unfortunately, a normal occurrence in those with PD, as over 68% of those with PD will fall at least once over the progression of their disease [8]. As falls are one of the leading causes of hospitalization, loss of independence, and increased mortality in this population, proper monitoring and prevention of falls is of utmost importance [9–12].

Currently, there is no accepted, standardized measure that holistically captures gait performance, which is of particular interest for populations with multifaceted gait impairments. As a result, the development of composite scores has gained attention because of their potential ability to incorporate multiple aspects of gait impairment into a single measure. The Gait Variability Index (GVI) was developed as a potential composite marker of spatiotemporal gait variability [13]. This score combines nine spatiotemporal variables often reported in the literature for gait instability or fall risk [13]. To avoid biasing the variables due to pace, Gouelle et al. used the absolute difference between consecutive values instead of using standard deviation or coefficient of variation [13]. Specifically, the GVI is calculated by weighting individual variables and comparing them with a healthy population index to create a *z*-score. Using a pathological population (i.e., Friedreich's ataxia), the GVI was able to differentiate between groups with and without gait disturbances [13]. However, Rennie et al. [14] questioned the validity of the GVI for individuals with PD when they observed GVI scores were similar between those with PD and healthy older adults, and the scores could not distinguish between mild and moderate disease severity. While the averages of some spatiotemporal variables during forward walking (e.g., stride time or stride length) may not be able to distinguish between those with and without PD [4, 15], it is not surprising to see similar measures of gait variability used in the calculation of the GVI. Specifically, the logarithmic calculation of the GVI introduced limitations in the interpretation; thus, the Enhanced Gait Variability Index (EGVI) was developed to take its place [16]. The EGVI is based upon three main alterations to the GVI: (1) solving the magnitude issue by adding a one before taking the natural log of the absolute distance between computed and reference values, (2) to solve the direction issue, the EGVI allows for values both above and below the standard score, and (3) the EGVI reduced redundancy between the variables by removing stride length, stride time, double support time, and swing time [16]. Using the remaining five parameters: step length, step time, stance time, single support time, and stride velocity, the EGVI computes the difference between successive values to determine the variation from one step to the next. Overall, the resulting EGVI score represents both the magnitude and variability of these five spatiotemporal parameters. An EGVI score of 100 signifies the amount of variability in a healthy population, and a 10-point difference corresponds to one standard deviation. An EGVI above 100 indicates greater gait variability than the healthy population, and an EGVI below 100 indicates less variability than the healthy population [16].

Although the EGVI is more strongly associated with functional outcomes in pathologic populations than the GVI, comparisons to its individual parts have yet to be studied. A single score may be recognized as a simple and effective way to communicate gait performance quickly in a clinical setting. For example, if appropriate cutoff scores can be established, a clinician may be able to identify a potential faller and intervene appropriately. Yet, it is still unknown if the EGVI score is more effective at predicting fall frequency than its individual parts. The purpose of the present study was to contrast the means of the individual spatiotemporal measures used as components of the EGVI as predictors of fall frequency and the EGVI as a single predictor of fall frequency in individuals with Parkinson's disease.

## 2. Materials and Methods

Individuals diagnosed with PD (i.e., those satisfying the United Kingdom Brain Bank Criteria for Parkinson Disease) were prospectively administered a gait evaluation as part of their routine clinical visit between August 2016 to May 2017. Diagnosis was confirmed by a fellowship-trained movement disorders neurologist.

A total of 273 patients (189M, 84F; Age: 68 ± 10) with idiopathic Parkinson's disease were included in the analysis. For participants with multiple visits and multiple gait assessments during the study period, data from a single gait assessment session were selected at random for inclusion in the analysis. This analysis was conducted as a retrospective review of consecutively collected clinical data. Patients were included in the analysis if they were able to independently complete the walking assessment without an assistive device. Data were collected while patients were in their self-reported best therapeutic state; active treatment with antiparkinsonian medication and deep brain stimulation were included. Best therapeutic state was chosen to capture the typical day-to-day walking state of those with PD. Patients who were “off” medication or off deep brain stimulation were excluded. Trials with freezes were also excluded. All participants provided written informed consent prior to participating, allowing their clinical data to be used for research purposes. A separate protocol was approved by the University's Institutional Review Board to conduct the retrospective review under the initial consent.

Overground gait performance was measured while patients walked at a comfortable, self-selected speed over an 8m Zeno Walkway (Zenometrics, Peekskill, NY). The instrumented walkway collected pressure data at 120 Hz during each of four passes. The walkway was centered within an isolated 12.2 m × 1.37 m collection hallway, free from distraction. A verbal cue was provided to begin walking, but no other cues were provided during the walks nor were any feedback provided regarding their performance. All patients started 1m before the walkway and turned around 1m after the end of the walkway. Data from the first and last 0.6 m of the walkway were excluded to eliminate the first and last steps on the mat to allow for the assessment of steady state walking. The pressure data were collected and processed using version c509.1 of the PKMAS software (ProtoKinetics, Havertown, PA) to extract spatial and temporal gait parameters including gait velocity, step length, step time, stance time, and single support time (Table 1). Processed data from all four passes were combined and averaged [16], resulting in a sufficient number of steps for analysis of gait variability in those with PD, based on the suggested step minimum of 40 steps [14, 18]. The EGVI is automatically computed by the PKMAS software using the five variables listed in Table 1. Specifically, it uses the absolute difference between consecutive values of the same series to come up with a composite score around a *z*-score of 100 (see Gouelle et al. [13, 16], for additional details).

A fellowship-trained movement disorders neurologist determined the patients modified Hoehn and Yahr (H&Y) staging. A verbal questionnaire was used to document the frequency of falls in the past three months, as seen in Parkinson's Foundation Quality Improvement Initiative [19]. Patients were categorized into five groups according to their reported frequency of falls (never, rarely, monthly, weekly, or daily), consistent with Parkinson's Foundation Quality Improvement Initiative.

One-way ANOVAs with Tukey's post hoc tests were used to identify differences in clinical and demographic metrics between the fall frequency groups. A multiple regression with the means of the individual spatiotemporal parameters that are used in the calculation of the EGVI (i.e., gait velocity, step length, step time, stance time, and single support time) was used to predict fall frequency. In an effort to maintain the clinical applicability, the regression model utilized the magnitudes (e.g., means) of the individual spatiotemporal components of the EGVI, rather than the step-to-step variability of those components. A linear regression was used to assess the relationship between the EGVI composite measure and fall frequency. The significance level for all tests was set at *p* < 0.05. All statistical tests were completed using SPSS 21 (IBM, Armonk, New York).

## 3. Results and Discussion

### 3.1. Results

Demographic and clinical scores across fall frequency groups can be found in Table 2, spatiotemporal parameters across fall frequency groups can be found in Table 3. The average number of steps analyzed for the sample was 44 steps, thus meeting the minimum recommended criteria for studying gait variability in those with PD [18]. Differences between fall frequency groups were only found for gait velocities, H&Y scores, and EGVI (*p* < 0.001). Post hoc tests revealed several significant differences between EGVI scores, mainly separating between two groups of fallers. While the no fall and rarely fall groups were not significantly different (*p* > 0.05), both the no fall group and the rarely fall group have significantly lower EGVI scores than both the monthly and daily fallers (*p* < 0.008). There were no significant differences between the monthly and daily fallers for the EGVI scores (*p* > 0.05). Similarly, the never fall group had lower H&Y scores than the monthly, weekly, and daily fall groups (*p* < 0.007), but no differences were observed between the monthly, weekly, and daily fall groups. Finally, the no falls group had greater gait velocity than the monthly or daily faller groups (*p* < 0.01), but no significant differences were observed between any other groups.

Although the regression model using the independent spatiotemporal components to predict fall frequency was significant (*F* (5, 267) = 3.987, *p*=0.002), none of the independent components were significant (gait velocity: *β* = −0.231, *t* = −0.469, *p*=0.640; step length: *β* = −0.053, *t* = −0.126, *p*=0.900; step time: *β* = −1.384, *t* = −0.637, *p*=0.525; stance time: *β* = 1.099, *t* = 0.626, *p*=0.532; and single support time: *β* = 0.468, *t* = 0.708, *p*=0.480). This model with individual components explained a total of 6.9% of the variance in fall frequency (*r* = 0.264). Alternatively, the regression using the EGVI (*β* = 0.389, *t* = 6.942) to predict fall frequency was significant (*F* (1, 271) = 48.193, *p* < 0.001) with an explained variance of 15.1% (*r* = 0.389).

### 3.2. Discussion

The purpose of this study was to assess the relationship between the mean values of the individual components of the EGVI, the EGVI composite measure, and falls in individuals with Parkinson's disease. Generally, it appears that the EGVI is a better predictor of an increased fall frequency in persons with PD than its individual spatiotemporal measures. Our data indicate that as EGVI increases, the frequency of reported falls also increases. Consistent with previous literature, this suggests patients who are falling more frequently have more variable gait, based on the interpretation of the EGVI.

Although the regression model using the mean values of the individual components of the EGVI to predict fall frequency was significant, the lack of significance for any of the model parameters supports the idea that a combination of measures may be more powerful to adequately quantify fall frequency. The model using the EGVI composite measure to predict fall frequency was significant and explained a small amount of the variability in fall frequency (15.1%). Variability in gait has been cited as a predictor of falls in those with PD as variability increases with disease progression and falls and reduces with levodopa medication [7]. Specifically, stride time variability measured with coefficient of variation has shown a slightly higher amount of explained variance in falls at 27% [7]. While the EGVI does predict more variability in fall frequency than the mean values of its individual parts, the amount of variability left to explain in this large, and representative sample is overwhelming. Predicting falls with high accuracy is extremely difficult in those with PD as numerous factors (e.g., age, disease duration, physical activity, cognition, and functional and clinical scores) have been shown to significantly predict falls, even in prospective studies [20–22]. While a single composite score may seem to be efficient and easy to use, it may leave too much variability unexplained, leading to incorrect or incomplete predictions for clinicians. Collectively, these results suggest that the ability of gait measures, including both the EGVI and its individual component parts, to predict fall frequency remains elusive.

While participants with a wide range of H&Y scores were included in this study, our sample of participants had an average H&Y score of 2–2.5, representative of a mild to moderately impaired PD population [23]. Interestingly, half of the individuals with PD evaluated (137 of the 273 participants) fell within 1 standard deviation of the healthy adult EGVI values reported by Gouelle et al. [16]. This is consistent with the findings of several other studies including mild to moderately impaired persons [4, 15], which found that measures of gait variability have been sensitive to differences between populations, while averages of these parameters are not [4, 24]. While sixteen participants exhibited an EGVI score below 100, 136 participants exhibited EGVI scores more than 1 SD above the norm (EGVI > 110) highlighting the wide range of EGVI scores in this sample. Although gait is more variable overall in this cohort of individuals (EGVI > 100), there is a large amount of intraindividual variability in the EGVI measure in this sample. As expected, patients who reported greater fall frequencies had greater disease severity ratings (i.e., H&Y scores), slower gait velocities, and greater EGVI scores. Yet, within fall groups, the standard deviation of EGVI scores is relatively consistent (between 11–14 points), demonstrating equal heterogeneity of gait among the groups. It should be noted that the small sample sizes in the weekly and daily faller groups (groups 3 and 4, respectively) are a limitation of this study. Frequent fallers may not be able to safely complete gait assessments and, perhaps more importantly, may self-select not to participate in potentially risky activities. Additionally, frequent fallers often utilize assistive devices, and those that regularly use assistive devices were excluded. Identifying and assessing these individuals may be beneficial to help better understand the gait profiles of frequent fallers.

While the EGVI provides an objective measure of gait variability, how to clinically interpret this score in order to prescribe specific interventions should be investigated further. Typical clinical gait assessments often rely on the magnitudes of spatiotemporal variables to make judgments about the quality of a patient's gait [23]. However, considering variability of temporal gait measures has been linked to falls in those with PD, examining individual measures of variability (e.g., standard deviation or coefficient of variation) and comparing them to the EGVI may provide additional information of better ways to predict fall frequency. Although the EGVI as a single predictor does not explain a large amount of variability in fall frequency, future studies may consider validating cutoff scores for different fall frequency groups. For example, a score could be developed to distinguish an appropriate cutoff score between the rarely and monthly fallers groups, since significant differences in the EGVI existed between these groups. When considering specific gait parameters to use in clinical gait assessments, it should be noted that the EGVI, like many gait measures, is subject to a large amount of variability among individuals with PD.

## 4. Conclusions

Although we observed a wide range of EGVI scores in this large cohort, patients who reported greater fall frequencies had greater EGVI scores, suggesting more variable gait. The results of this investigation indicate that the EGVI is a slightly better predictor of increased fall frequency in individuals with PD than the individual spatiotemporal components. Future investigations predicting fall frequency in individuals with PD should expand upon this work by including both composite measures and assessing intraindividual variability.

## Figures and Tables

**Table 1:** Definitions of the five parameters used in the Enhanced Gait Variability Index calculation.

Parameters	Definition
Gait velocity	Sum of all stride lengths divided by the sum of all stride times (m/s)
Step length	Distance between successive heel points of opposite feet, measured parallel to the direction of progression (m)
Step time	Time take between the first contact of one foot to the first contact of the following contralateral foot (s)
Stance time	Time when foot is in contact with the ground (s)
Single support time	Time when only the evaluated foot is in contact with the ground without contact from the contralateral foot (s)

Note: based on Huxham et al. [17].

**Table 2:** Clinical and demographic metrics across fall frequency groups.

	EGVI		Hoehn & Yahr score		Age (years)
	Mean ± SD	(min, max)	Mean ± SD	(min, max)	Mean ± SD
Fall score: 0 (*n* = 186)	110 ± 11	(95, 144)	2.0 ± 0.5	(1, 4)	68 ± 10
Fall score: 1 (*n* = 47)	113 ± 11	(94, 137)	2.2 ± 0.6	(1, 3)	68 ± 8
Fall score: 2 (*n* = 24)	123 ± 14	(100, 149)	2.6 ± 0.5	(1.5, 4)	65 ± 11
Fall score: 3 (*n* = 8)	119 ± 12	(100, 135)	2.7 ± 0.7	(2, 4)	68 ± 9
Fall score: 4 (*n* = 8)	130 ± 14	(102, 150)	2.8 ± 0.8	(2, 4)	65 ± 5
Total (*n* = 273)	112 ± 12	(94, 150)	2.1 ± 0.6	(1, 4)	68 ± 10

Fall scores: 0, no falls; 1, rarely falls; 2, monthly falls; 3, weekly falls; 4, daily falls. EGVI: Enhanced Gait Variability Index.

**Table 3:** Mean and standard deviations of spatiotemporal parameters across fall frequency groups.

	Velocity (m/s)	Step length (m)	Step time (s)	Stance time (s)	Single support time (s)
Fall score: 0 (*n* = 186)	1.10 ± 0.21	0.61 ± 0.10	0.57 ± 0.06	0.75 ± 0.09	0.39 ± 0.03
Fall score: 1 (*n* = 47)	1.05 ± 0.25	0.59 ± 0.12	0.57 ± 0.07	0.76 ± 0.10	0.39 ± 0.04
Fall score: 2 (*n* = 24)	0.92 ± 0.20	0.53 ± 0.12	0.59 ± 0.07	0.80 ± 0.11	0.38 ± 0.05
Fall score: 3 (*n* = 8)	1.03 ± 0.13	0.59 ± 0.05	0.57 ± 0.06	0.76 ± 0.09	0.39 ± 0.05
Fall score: 4 (*n* = 8)	0.84 ± 0.19	0.51 ± 0.09	0.62 ± 0.10	0.85 ± 0.15	0.40 ± 0.06
Total (*n* = 273)	1.06 ± 0.22	0.57 ± 0.06	0.60 ± 0.11	0.76 ± 0.1	0.39 ± 0.04

Fall scores: 0, no falls; 1, rarely falls; 2, monthly falls; 3, weekly falls; 4, daily falls. Data provided are averaged between left and right limbs.

## Data Availability

The retrospective gait data from the Interdisciplinary Florida Registry and Movement Disorders database used to support the findings of this study may be released upon application to the UF Health Fixel Institute for Neurological Diseases and the University of Florida Institutional Review Board.

## References

[1] Hassan A., Wu S. S., Schmidt P. (2012). What are the issues facing Parkinson’s disease patients at ten years of disease and beyond?: data from the NPF-QII study. *Parkinsonism & Related Disorders*.

[2] Ebersbach G., Moreau C., Gandor F., Defebvre L., Devos D. (2013). Clinical syndromes: Parkinsonian gait. *Movement Disorders*.

[3] Morris M. E., Huxham F., McGinley J., Dodd K., Iansek R. (2001). The biomechanics and motor control of gait in Parkinson disease. *Clinical Biomechanics*.

[4] Hausdorff J. M., Cudkowicz M. E., Firtion R., Wei J. Y., Goldberger A. L. (1998). Gait variability and basal ganglia disorders: stride-to-stride variations of gait cycle timing in Parkinson’s disease and Huntington’s disease. *Movement Disorders*.

[5] Nanhoe-Mahabier W., Snijders A. H., Delval A. (2011). Walking patterns in Parkinson’s disease with and without freezing of gait. *Neuroscience*.

[6] Blin O., Ferrandez A. M., Serratrice G. (1990). Quantitative analysis of gait in Parkinson patients: increased variability of stride length. *Journal of the Neurological Sciences*.

[7] Schaafsma J. D., Giladi N., Balash Y., Bartels A. L., Gurevich T., Hausdorff J. M. (2003). Gait dynamics in Parkinson’s disease: relationship to Parkinsonian features, falls and response to levodopa. *Journal of the Neurological Sciences*.

[8] Wood B. H., Bilclough J. A., Bowron A., Walker R. W. (2002). Incidence and prediction of falls in Parkinson’s disease: a prospective multidisciplinary study. *Journal of Neurology, Neurosurgery & Psychiatry*.

[9] Stolze H., Klebe S., Zechlin C., Baecker C., Friege L., Deuschl G. n. (2004). Falls in frequent neurological diseases. *Journal of Neurology*.

[10] Bloem B. R., Grimbergen Y. A. M., Cramer M., Willemsen M., Zwinderman A. H. (2001). Prospective assessment of falls in Parkinson’s disease. *Journal of Neurology*.

[11] Allen N. E., Schwarzel A. K., Canning C. G. (2013). Recurrent falls in Parkinson’s disease: a systematic review. *Parkinson’s Disease*.

[12] Matinolli M., Korpelainen J. T., Sotaniemi K. A., Myllylä V. V., Korpelainen R. (March 2011). Recurrent falls and mortality in Parkinson’s disease: a prospective two-year follow-up study. *Acta Neurologica Scandinavica*.

[13] Gouelle A., Mégrot F., Presedo A., Husson I., Yelnik A., Penneçot G.-F. (2013). The gait variability index: a new way to quantify fluctuation magnitude of spatiotemporal parameters during gait. *Gait & Posture*.

[14] Rennie L., Dietrichs E., Moe-Nilssen R., Opheim A., Franzén E. (2017). The validity of the Gait Variability Index for individuals with mild to moderate Parkinson’s disease. *Gait & Posture*.

[15] Crenna P., Carpinella I., Rabuffetti M. (2007). The association between impaired turning and normal straight walking in Parkinson’s disease. *Gait & Posture*.

[16] Gouelle A., Rennie L., Clark D. J., Mégrot F., Balasubramanian C. K. (2018). Addressing limitations of the Gait Variability Index to enhance its applicability: the enhanced GVI (EGVI). *PLoS One*.

[17] Huxham F., Gong J., Baker R., Morris M., Iansek R. (2006). Defining spatial parameters for non-linear walking. *Gait & Posture*.

[18] Rennie L., Löfgren N., Moe-Nilssen R., Opheim A., Dietrichs E., Franzén E. (2018). The reliability of gait variability measures for individuals with Parkinson’s disease and healthy older adults—the effect of gait speed. *Gait & Posture*.

[19] Okun M. S., Siderowf A., Nutt J. G. (2010). Piloting the NPF data-driven quality improvement initiative. *Parkinsonism & Related Disorders*.

[20] Custodio N., Lira D., Herrera-Perez E. (2016). Predictive model for falling in Parkinson disease patients. *eNeurologicalSci*.

[21] Allcock L. M., Rowan E. N., Steen I. N., Wesnes K., Kenny R. A., Burn D. J. (2009). Impaired attention predicts falling in Parkinson’s disease. *Parkinsonism & Related Disorders*.

[22] Yarnall A., Rochester L., Burn D. J. (2011). The interplay of cholinergic function, attention, and falls in Parkinson’s disease. *Movement Disorders*.

[23] Hass C. J., Malczak P., Nocera J. (2012). Quantitative normative gait data in a large cohort of ambulatory persons with Parkinson’s disease. *PLoS One*.

[24] Hausdorff J. M., Rios D. A., Edelberg H. K. (2001). Gait variability and fall risk in community-living older adults: a 1-year prospective study. *Archives of Physical Medicine and Rehabilitation*.

